# 3D Freehand Ultrasound Imaging of Optic Nerve Sheath

**DOI:** 10.1097/RLI.0000000000001250

**Published:** 2025-11-26

**Authors:** Kai Riemer, Nora Zarranz Bozal, Hendrik Mugele, Giovanni Vinetti, Jay Carr, Connor Howe, Giacomo Strapazzon, Justin Steven Lawley

**Affiliations:** Institute of Mountain Emergency Medicine Bolzano Italy (K.R., N.Z.B., G.V., J.C., G.S., J.S.L.); Institute of Sport Science, Innsbruck University, Innsbruck, Austria (H.M., C.H., J.S.L.)

**Keywords:** optic nerve sheath diameter, ONSD, intracranial pressure, icp, 3D ultrasound, volumetric ultrasound, neurology

## Abstract

**Objectives:**

The optic nerve sheath expands with elevated intracranial pressure, and its diameter is a sensitive proxy measure. While 2D transorbital ultrasound is well-established for detecting elevated intracranial pressure (>20 mm Hg), it shows limited sensitivity to modest or gradual changes, potentially due to geometric assumptions and imaging misalignment. This study introduces freehand 3D ultrasound imaging of the optic nerve sheath to reduce measurement ambiguity and enhance the fidelity of optic nerve sheath assessment as a noninvasive marker of intracranial pressure.

**Materials and Methods:**

Twelve healthy participants (28.7 ± 8.2 y; 6 females) underwent freehand 3D optic nerve sheath imaging during 24 hours of normobaric hypoxia in an environmental chamber (FiO_2_ = 13.1%). Scans were acquired in a head-raised (30 degrees) position at baseline, 6, 12, and 24 hours. Cardiorespiratory parameters, cerebral blood flow, and end-tidal gases were recorded. In 6 participants, postural effects were also assessed at baseline. Nutrition and hydration were standardized to 75% of daily requirements.

**Results:**

The optic nerve sheath’s curved, noncircular shape makes the 2D diameter measurement of the sheath a geometric mismatch and an inadequate descriptor. Probe misalignments of 1.2 mm or 15 degrees led to 2D diameter errors (9%/18.7%) exceeding observer variability (2.6%/3.4%). Freehand 3D imaging was reproducible and yielded more informative metrics, including sheath area and thickness. Internal sheath thickness was more responsive to posture and hypoxia than internal 2D diameter (+31.5%, *P* = 0.01 vs +2.4%, *P* = 0.9; +8.2%, *P* = 0.04 vs +1.8%, *P* = 0.9). A time effect was observed for internal sheath thickness (*P* = 0.04), and both area and thickness correlated with internal carotid artery velocity (area: r = 0.9, *P* = 0.05; thickness: r = 0.8, *P* = 0.07), consistent with physiological expectations.

**Conclusion:**

By eliminating geometric assumptions and misalignment, freehand 3D ultrasound improves the fidelity and sensitivity of optic nerve sheath measurements at the bedside.

The optic nerve sheath is an extension of the cranial dura mater that envelops the optic nerve. Its subarachnoid space communicates freely with intracranial cerebrospinal fluid spaces, meaning that changes in intracranial pressure (ICP) are directly transmitted to the sheath surrounding the optic nerve. As ICP rises, the cerebrospinal fluid within the sheath causes it to distend, increasing the optic nerve sheath diameter (ONSD).^1^ This anatomic continuity makes ONSD a sensitive indirect marker of ICP, measurement of which improves patient outcome in conditions like traumatic brain injury, hydrocephalus, and intracranial hypertension.^2–4^ Environmental hypoxia, a condition similar to hypoxia observed in critically ill patients, is associated with elevated ICP and offers a safe model to study optic nerve sheath changes.

ONSD is a good binary predictor of intracranial hypertension.^1^ In the moderate ICP range, ONSD changes linearly and dynamically with ICP.^5,6^ However, at low levels of ICP and when changes evolve gradually, as seen in environmental hypoxia, sheath expansion is often obscured by measurement variability. At high ICP, sheath diameter plateaus.^7,8^ Notably, the optic nerve sheath is neither cylindrical nor uniform.^9^ As such, a single diameter measurement may represent both an inadequate descriptor and a geometric mismatch. The optic disc is not a consistently identifiable landmark; its appearance varies highly across imaging planes, which makes fixed-distance measurements ambiguous. The shape of the optic nerve also changes with eye movement. Together, these factors may help explain ONSD’s limited sensitivity to modest (12 to 20 mm Hg) and gradually evolving changes.

Bedside measurement of ONSD involves imaging 3- to 8 mm behind the globe and measuring the sheath’s internal and external diameter,^10^ typically through transorbital ultrasound. To address the geometric complexities, measuring orthogonal planes^11^ or estimating sheath area using elliptical approximations have been investigated; but these rely on simplified assumptions.^12^ Coronal-view^13,14^ and C-scan ultrasound techniques^15–17^ offer direct, perpendicular measurements with good agreement to CT and MRI, but are limited by uncertain alignment with the optic nerve’s anatomic axis and absence of true volumetric information. While volumetric ophthalmic imaging using customized 3D probes has been demonstrated,^18^ these systems are not widely accessible, and their volumetric data have not been leveraged. This, however, is important, as MRI studies suggest that volumetric assessment of the optic nerve sheath is more sensitive to ICP related changes than 2D diameter measurements.^19^ MRI cannot be used at the bedside, but freehand 3D ultrasound can, providing volumetric assessment of the optic nerve sheath with greater practicality and higher resolution using a standard ultrasound scanner.

In this study, we test 3 hypotheses: first, that freehand 3D ultrasound can reliably capture the optic nerve sheath at the bedside; second, that measurement inaccuracies are primarily driven by probe alignment and anatomic ambiguity; and third, that 3D imaging yields more sensitive metrics of sheath morphology and its physiological correlates when changes in optic nerve sheath dimensions are moderate and gradual. These hypotheses are evaluated through postural shift experiments and during a 24-hour acute hypoxic exposure in healthy volunteers.

## MATERIALS AND METHODS

### Ethical Approval

The study was approved by the Ethics Committee of the Autonomous Province of Bolzano (No. 92-2020, Opinion 42-2024, June 27, 2024) and conducted in accordance with the Declaration of Helsinki. All participants gave written informed consent after receiving oral and written information before the start of the study.

### Study Design

**FIGURE 1:**
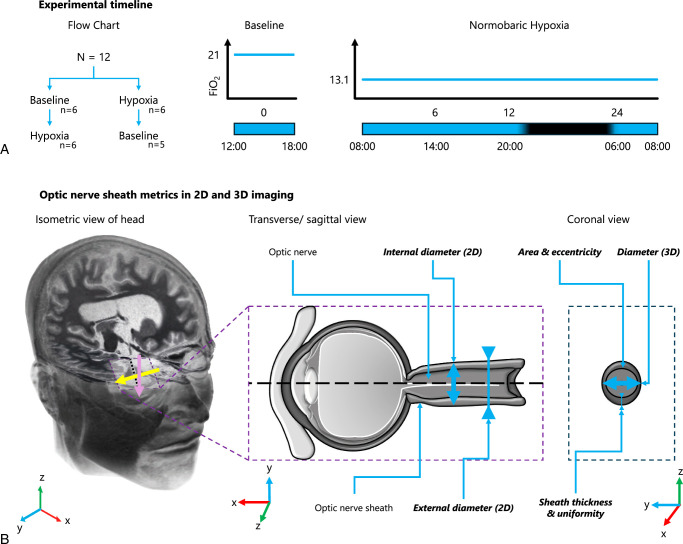
Experimental timeline and measurement metrics (bold) of the study. A, Participants attended 2 separate visits: baseline (FiO_2_ = 21%) and 24-hour exposure to normobaric hypoxia (FiO_2_ = 13.1%), including an overnight stay. The order of visits was randomized but equally distributed, and only one baseline per participant was acquired. B, Optic nerve sheath metrics used in 2D and 3D (bold, italic). Internal and external diameters (2D) were obtained from the transverse and sagittal views. Diameter (3D), area, eccentricity, sheath thickness, and uniformity were obtained from the coronal view.

This prospective, repeated-measures study included a baseline visit and a separate 24-hour normobaric hypoxia exposure, both conducted in an environmental chamber (terraXcube, Italy; altitude 262 m). An overview of the experimental timeline and organization is shown in Figure 1A. Ambient conditions were controlled: normobaric hypoxia was simulated with a fraction of inspired oxygen (FiO_2_) set to 13.1%; temperature at 21 °C (day; 5:00 am to 10:00 pm) and 20 °C (night; 10:00 to 5:00 am), pressure at 995 mbar, and relative humidity at 50%. A maximum of 4 participants were present in the chamber at a time. During the baseline visit, the optic nerve sheath was measured in the head-raised position (30 degrees) in all 12 participants, and in 6 of them, additional measurements were performed in the supine and seated positions. During the 24-hour hypoxia exposure, all optic nerve sheath measurements were performed only in the head-raised position (30 degrees) and taken at 6, 12, and 24 hours. Cardiorespiratory and cerebral blood flow measurements were also done in the head-raised (30 degrees) position at all times. The order of sessions was randomized with equal number of participants and a minimum 24-hour washout period if baseline followed exposure. Food and fluid intake were standardized to ∼75% of estimated energy expenditure and total daily fluid requirements. Participants received a light meal one hour before entry, sandwiches at 10:00 am and 5:00 pm, 2 fruits, 2 energy bars, water, and tea every 6 hours, consumed ad libitum. Participants slept without a blanket.

### Subject Recruitment and Screening

Twelve participants completed the 24-hour hypoxia exposure, but one participant missed the baseline assessment. An additional participant was included exclusively for the posture assessment. The participant demographics are summarized in Table 1. Eligibility (age: 18 to 50 y, BMI: <30, residing below: 1500 m) was confirmed through medical examination, including medical history, arterial oxygen saturation, and screening for acute mountain sickness, conducted by a qualified MD. Exclusion criteria included acute or chronic medical conditions (cardiovascular, respiratory, neurological, metabolic), pregnancy, smoking, recent high-altitude exposure (>2500 m within 4 wk), substance abuse, Raynaud’s disease, recent surgery or blood donation (<6 mo), and recent cold-acclimatization activities. Female participants underwent a pregnancy urine test strip, and participants abstained from alcohol and exercise for 24 hours before measurements.

**TABLE 1:** Demographics of the Study Participants (n = 12)

Characteristic	Value
No.participants	12
Sex (male/ female)	6/ 6
Age (y)	28.7±8.2
Height (m)	1.8±0.1
Weight (kg)	71.48±12.45
BMI (kg/m^2^)	22.7±3.1
BSA (m^2^)	1.9±0.2

Values are presented as mean±SD or frequency, as appropriate.

BMI indicates body mass index; BSA, body surface area.

### Ultrasound Imaging of Optic Nerve Sheath

The optic nerve sheath of the right eye was assessed. Each body position was maintained for 5 minutes before imaging. At each time point, 2D ultrasound images were acquired in transverse and sagittal planes. Freehand 3D acquisitions were performed by sweeping along the *z*-axis (forehead to chin) or *y*-axis (nose to right temple), as shown in Figure 1B (pink/ yellow arrow). All subsequent analysis was performed only on acquisitions obtained in the z-direction (transverse oriented sweeps). Participants were instructed to look straight forward with a fixed gaze. Ultrasound gel was applied liberally, and the transducer was positioned over the right eye without direct contact. To minimize pressure and distortion, the operator’s hand was supported on a rigid surface such as the nose, midface, or forehead. Imaging was performed with a Philips CX50 scanner (L12-3 linear probe, 9.75 MHz centre frequency, 52 Hz frame rate, MI <0.1). Recordings were captured through screen recording (USB 3.0 Video Capture Device, StarTech). Probe position and orientation were acquired with a Gen3 motion tracking system (AMFITRACK). Imaging and tracking data were synchronously streamed to a custom Python script. A coordinate transformation was applied such that the initial probe orientation defined the reference frame for both the tracking system and the ultrasound images. Position data were smoothed using a Savitzky–Golay filter. Three-dimensional volumes were reconstructed by mapping 2D images into 3D space with weighted voxel intensities averaged over a 1 mm elevational thickness. The voxel size of each volume was 0.044 mm. Acquisition was repeated a minimum of 3 times. The full 3D reconstruction pipeline, including visualization and alignment, along with an example data set and Jupyter notebook, is available (at: https://github.com/KR616/3D_freehand_optic_nerve_sheath.git).

### Optic Nerve Sheath Measurements

Figure 1B illustrates the 5 metrics used to characterize the optic nerve sheath: 2D diameter, 3D diameter, area, sheath thickness, uniformity, and eccentricity. The 2D diameter was taken from the central or optimal slice of the 3D volume. All other metrics were derived from segmentations of the coronal view. Mean diameter was calculated by placing 100 equidistant nodes along the sheath contour and averaging distances between opposing node pairs. Sheath thickness was computed by segmenting both the nerve and sheath and projecting 360 radial lines, then averaging their distance. Uniformity was defined as the SD of sheath thickness across 360 degrees, sampled at 1-degree intervals. Lower values indicate a more homogeneous sheath thickness. Eccentricity was obtained from an ellipse fitted to the sheath segmentation.

Images were acquired by a single operator (K.R.), who also manually aligned the optic nerve along its axis of symmetry. Two operators (K.R. and N.Z.B.) independently performed all measurements, blinded and in random order, using the same image sets. Operators could adjust the image dynamic range and repeated each measurement 3 times; the average of these means was used for analysis. The conventional diameter was measured at 3 (Figs. 4) or 4 mm (Table 3, Figs. 5, 6) below the optic disc. Proposed metrics were assessed using the mean projection from 3 to 5 mm. All metrics were applied separately to internal and external sheath contours.

### Cardiorespiratory and Cerebral Blood Flow Measurements

Time average blood flow velocity of the right internal carotid and left vertebral arteries was measured with an 15L4 linear array by continuous duplex vascular sonography (uSmart 3300, Terason). Heart rate was measured continuously with a 3-lead ECG system, while continuous blood pressure was obtained through photoplethysmography (Finometer, Finapres Medical Systems). The peripheral saturation of oxygen (SpO_2_) was assessed through forehead oximetry (Maxfast Nellcor, Medtronic). End tidal CO_2_ and O_2_ were measured with a respiratory gas analyser (ADInstruments).

### Statistics

To assess probe alignment, the percentage difference between the reference plane and the measurement plane was determined. To assess intraobserver repeatability, the median coefficient of variation (CV) was used:


(1) $$\mathrm{CV}=\frac{\mathrm{SD}}{\mathrm{Mean}}100\%$$

To assess the interobserver variability, the median percentage difference between observers across participants and time points was used.


(2) $$∆\%=\frac{|O1-O2|}{\frac{O1+O2}{2}}100\%$$

where *O1* and *O2* represent the mean of the 3 repeated runs for observer 1 and observer 2, respectively. To further evaluate interobserver agreement, the intraclass correlation coefficient was computed.^20^ ICC considers both the variance originating from differences between subjects and the variance originating from measurement error.


(3) $$\mathrm{ICC}=\frac{\mathrm{Between}-\mathrm{subject variance}}{\mathrm{Between}-\mathrm{subject variance}+\mathrm{Error Variance}}$$

A two-way random-effects, absolute-agreement model. Both single-measure ICC [ICC(A,1)] and average-measure ICC [ICC(A,2)] were calculated. ICC(A,1) reflects the reliability of a single observer’s measurement, whereas ICC(A,2) reflects the reliability of the average of both observers. Interpretation followed established guidelines: values <0.5 were considered poor, 0.5 to 0.75 moderate, 0.75 to 0.9 substantial, and ≥0.9 excellent reliability. To assess the level of agreement between the 2D and 3D diameters and between the internal and the external diameters, Bland-Altman analysis was used. To evaluate the smallest detectable morphometric changes beyond measurement error, the minimum detectable change was calculated.^20^


(4) $$\mathrm{MDC}=1.96\;\sqrt{2}\mathrm{wSD}$$

The within-subject SD (wSD) was computed across 3 repeated runs at each time point. This procedure was repeated across all participants, and the resulting MDC values were averaged. To provide a conservative estimate that captures both intraobserver and interobserver variability, results were pooled across the 2 observers. To assess changes in metrics during normal shifts in gravitational gradients, a repeated-measures analysis of variance (ANOVA) was performed. To evaluate changes in metrics over time during hypoxia, linear mixed-effects models were fitted for each outcome variable, with time as a fixed effect and participant as a random effect (random intercept), accounting for repeated measures. Physiological parameters were included as fixed covariates. Models were estimated using restricted maximum likelihood. Correlation analyses (Pearson r) were conducted to assess the linear relationship between each metric and physiological parameters. Statistical significance was defined as *P* <0.05.

## RESULTS

### Three-dimensional Freehand Ultrasound Scan of Optic Nerve Sheath

**FIGURE 2:**
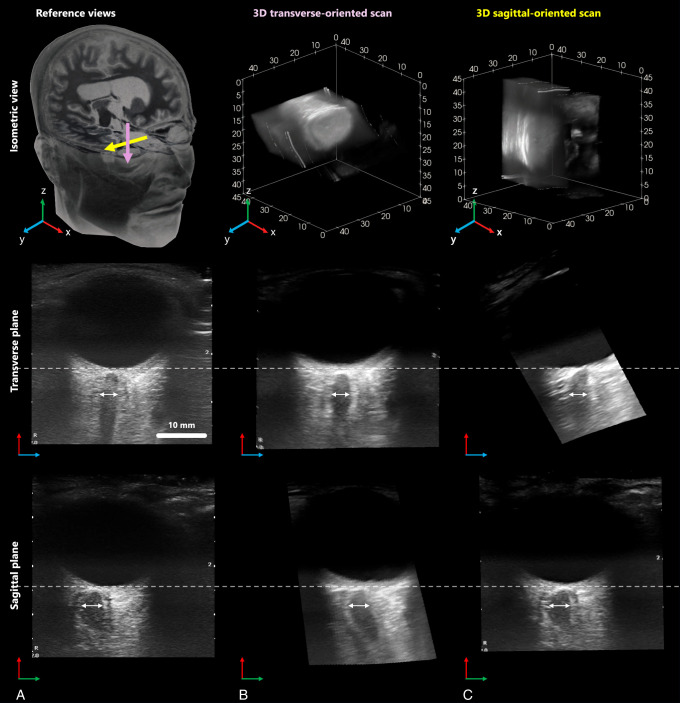
Three-dimensional freehand ultrasound scans of the optic nerve sheath. Column (A) shows the reference scan, column (B) the transversely oriented scan acquired along the *z*-axis (forehead to occiput), and column (C) the sagittally oriented scan acquired along the *y*-axis (nose to temporal region). The top row displays isometric views of the reconstructed 3D volumes, the middle row shows transverse slices at the central plane, and the bottom row shows sagittal slices. White arrows of equal length indicate the spatial comparability across scan orientations. Images have been rotated for visualization purposes.

Figure 2 shows three-dimensional freehand ultrasound scans of the optic nerve sheath, color-coded and arranged in 3 columns: (A) a reference scan, (B) a transverse scan acquired along the *z*-axis (forehead to occiput), and (C) a sagittal scan acquired along the *y*-axis (nose to temporal region). Each row represents a different view of the 3D reconstruction: the top row shows an isometric view, the middle row a central transverse slice, and the bottom row a central sagittal slice. White lines and arrows of equal length highlight spatial comparability between orientations. The lower resolution in the elevational direction results in visible blurring, especially in planes perpendicular to the sweep. Views are rotated to visually align the different orientations. Supplementary video 1 (Supplemental Digital Content 1, http://links.lww.com/RLI/B74) shows an animated 3D reconstruction.

### Coronal View and Coronal Projection of Optic Nerve Sheath

**FIGURE 3:**
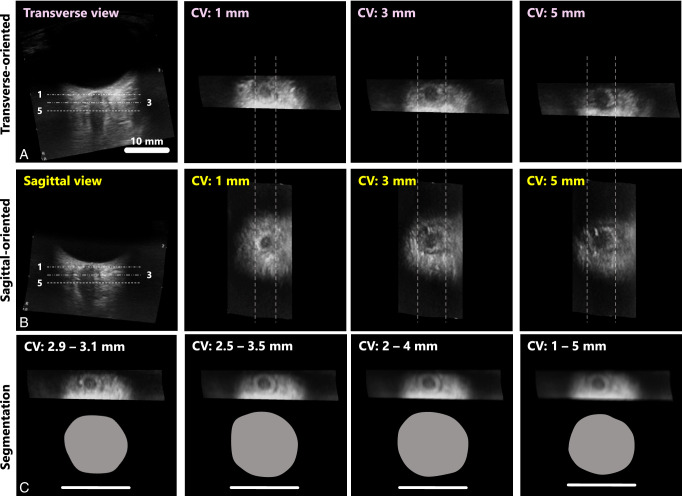
Coronal view (CV) and projection images of the optic nerve sheath. A, Shows coronal slices from a transversely oriented 3D ultrasound scan. B, Shows slices from a sagittally oriented scan. C, Displays mean intensity projection images derived from the transversely oriented scan. In rows (A) and (B), the first column represents the reference viewing plane, followed by coronal views at 1 mm, 3 mm, and 5 mm depths below the optic disc. In row (C), projection images are shown with increasing projection thickness: ±0.1 mm (2.9 to 3.1 mm), ±0.5 mm (2.5 to 3.5 mm), ±1 mm (2 to 4 mm), and ±2 mm (1 to 5 mm).

Figure 3 shows coronal views of the optic nerve sheath from 2 scan orientations and various projection depths, using the same colour code as Figure 2. The coronal view is animated in supplementary video 2 (Supplemental Digital Content 2, http://links.lww.com/RLI/B75). Row (A) displays coronal slices from a transverse scan, and row (B) from a sagittal scan. Row (C) shows projection images from the transverse scan at increasing thicknesses: ±0.1 mm, ±0.5 mm, ±1 mm, and ±2 mm. In rows (A) and (B), the first column shows the reference plane, followed by slices at 1 mm, 3 mm, and 5 mm below the optic disc. Projection images in row (C) represent mean intensity projections across the specified depth ranges. These projections summarize sheath volume rather than showing a single slice. As projection width increases, clarity improves initially but diminishes with excessive depth averaging. Manual segmentations of the internal sheath (gray shape, enlarged) are shown for each image, highlighting changes in boundary definition evolving with depth and confirming the sheath’s noncircular cross-section. The white lines are included for visual reference.

### Sensitivity of Optic Nerve Sheath Diameter to Probe Position and Orientation

**FIGURE 4:**
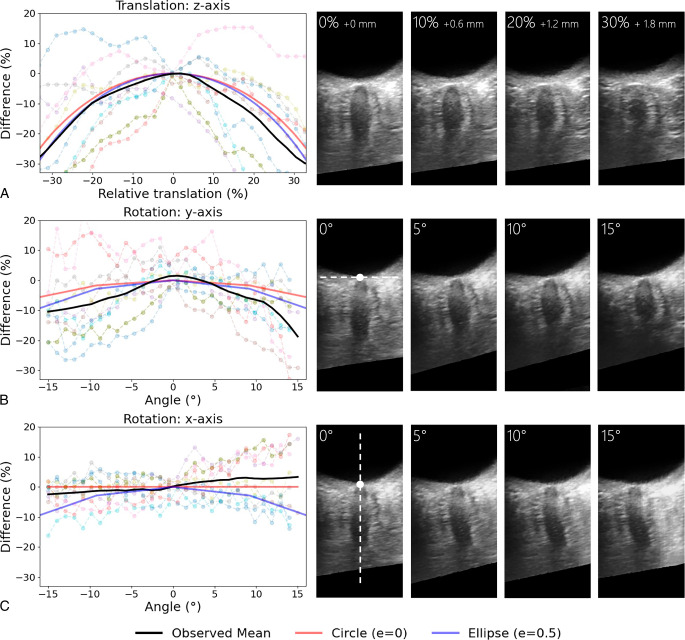
Sensitivity of optic nerve sheath diameter to probe position and orientation. Plots show the difference in measured diameter 3 mm below the optic disc resulting from (A) probe translation (forehead to occiput), (B) pitch rotation around the transverse axis, and (C) roll rotation around the longitudinal axis. Black line shows the mean of the measurement, red line the expected values if the optic nerve sheath was round, and blue line shows the expected values for an ellipse (e = 0.5). White lines indicate the axis of rotation. These examples illustrate how small deviations in probe alignment can lead to substantial variability in diameter measurements, even when images can be considered high quality.

The sensitivity of optic nerve sheath diameter measurements to variations in probe position and orientation is illustrated in Figure 4 and supplementary video 3 (Supplemental Digital Content 3, http://links.lww.com/RLI/B76). Row (A) shows the effect of probe translation along the *z*-axis (superior to inferior direction), while rows (B) and (C) show the effects of probe rotation around the *y*-axis and *x*-axis, respectively. Each plot is accompanied by representative transverse images. The black line represents the mean observation difference across participants. The red line shows the expected difference assuming a circular and the blue line an elliptical (e = 0.5) optic nerve sheath. Group-level data from 11 participants show a mean difference of 5.7% at 10% translation (relative to manually observed reference diameter at 3 mm), increasing to 13.3% at 20% and 27.8% at 30%. A 10-degree rotation around the *y*-axis resulted in a 6.4% difference, rising to 18.7% at 15 degrees. Rotation around the *x*-axis produced differences ranging from 2% to 3.6% across the same angular range. In the presented example, the percentage translations are accompanied by physical values. A 10% (0.6 mm) translation along the *z*-axis caused a 5.3% difference, which increased to 9% at 1.2 mm (20%) and 21.9% at 1.8 mm (30%). Notably, identifying the exact location of the optic disc is ambiguous, as it spans different depths across imaging planes and appears thinnest in the central row B.

### Intraobserver and Interobserver Variability and Comparison With 2D Measurement

**FIGURE 5:**
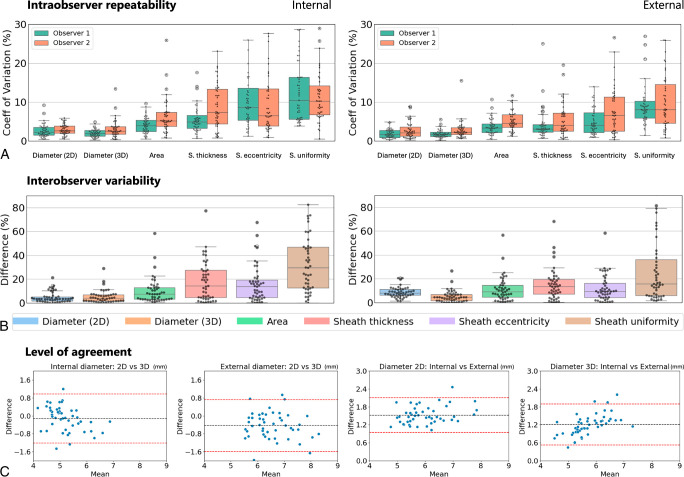
Repeatability and agreement of 2D and 3D metrics. A, Intraobserver repeatability is shown as the coefficient of variation for each metric across 3 repeated measurements and for 2 observers. B, Interobserver variability is expressed as the median percent difference between observers. C, Bland Altman plot compares the 2D level of agreement between 2D and 3D diameters and between internal and external diameters. With increasing complexity, the coefficient of variation/ difference increased.

Figure 5 Supplementary video 4 (Supplemental Digital Content 4, http://links.lww.com/RLI/B77) shows 3 separate 3D freehand acquisitions for visual comparison. In Figure 3, intraobserver and interobserver repeatability and variability are shown. For internal measurements, the most repeatable metric between the two observers was diameter (3D) (CV%: 1.9/ 2.6%), followed by diameter (2D) (CV%: 2.3/ 2.6%) and area (CV%: 4/5.2%). Sheath thickness (CV%: 4.9/7.4%), eccentricity (CV%: 6.5/8.6%), and uniformity (CV%: 10.3/10.5%) had higher intraobserver variability. For external measurements, diameter (3D) again showed the lowest repeatability (CV%: 1.7% to 2.3%), followed by diameter (2D) (CV%: 1.6% to 2.1%) and area (CV%: 3.3% to 4.6%). Interobserver variability showed a consistent pattern across metrics [Δ% + ICC(A,1) + ICC(A,2)]. For internal measurements, interobserver differences were lowest for diameter [2D: Δ%=3.4%, ICC(A,1)=0.89, ICC(A,2)=0.94; 3D: Δ%=4.0%, ICC(A,1)=0.80, ICC(A,2)=0.89] and area [Δ%=7.4%, ICC(A,1)=0.81, ICC(A,2)=0.89]. Thickness [Δ%=14.4%, ICC(A,1)=0.78, ICC(A,2)=0.88] and eccentricity [Δ%=13.8%, ICC(A,1)=0.72, ICC(A,2)=0.84] showed moderate variability, while Uniformity showed the largest variability [Δ%=30.5%, ICC(A,1)=0.43, ICC(A,2)=0.60]. For external measurements, variability was again best for diameter [2D: Δ%=8.0%, ICC(A,1)=0.75, ICC(A,2)=0.86; 3D: Δ%=4.5%, ICC(A,1)=0.85, ICC(A,2)=0.92] and area [Δ%=9.2%, ICC(A,1)=0.83, ICC(A,2)=0.92]. Thickness [Δ%=13.5%, ICC(A,1)=0.84, ICC(A,2)=0.91] and eccentricity [Δ%=9.4%, ICC(A,1)=0.72, ICC(A,2)=0.84] showed moderate variability, while Uniformity remained poorest [Δ%=19.1%, ICC(A,1)=0.46, ICC(A,2)=0.63]. Bland-Altman analysis showed agreement between 2D Diameter and 3D derived metrics [bias (SD, LoA) in mm]: internal diameter (2D) versus diameter (3D): –0.1 (0.6, –1.2 to 1); external diameter (2D) versus diameter (3D): –0.4 (0.6, –1.6 to 0.7); internal versus external diameter (2D): 1.5 (0.3, 0.9 to 2.1); internal versus external diameter (3D): 1.2 (0.3, 0.5 to 1.9).

### Minimum Detectable Change

Table 2 summarizes the minimum detectable change (MDC) for internal and external morphometric measurements. Metrics that required the least manual input, such as 2D and 3D diameters, had the lowest relative MDC values (7.6% and 7.1% internally; 6.5% and 6.3% externally). Area and sheath thickness showed intermediate MDC values (14.5% and 18.3% internally; 12.4% and 11.7% externally), while eccentricity and uniformity had the highest MDC values (25.8% and 39.6% internally; 16.6% and 32.0% externally). The overall ranking of metrics was consistent between internal and external approaches, with external measurements showing slightly lower MDC% for area and sheath thickness. While MDC values were smaller when calculated for each observer individually, pooled estimates incorporate both intraobserver and interobserver error and may better reflect real-world application.

**TABLE 2:** Minimum Detectable Change (MDC) for Internal and External Morphometric Measurements

Metric	Units	Mean	wSD	MDC	MDC%
Diameter (2D)	mm	5.25/6.77	0.14/0.16	0.40/0.44	7.6%/6.5
Diameter (3D)	mm	5.13/6.34	0.13/0.14	0.37/0.40	7.1%/6.3
Area	m^2^	20.30/31.10	1.06/1.39	2.94/3.86	14.5%/12.4
Sheath thickness	mm	0.85/1.41	0.06/0.06	0.16/0.16	18.3%/11.7
Sheath eccentricity	1	0.54/0.62	0.05/0.04	0.14/0.10	25.8%/16.6
Sheath uniformity	mm	0.35/0.35	0.05/0.04	0.14/0.11	39.6%/32.0

Values represent the observer pooled mean, within-subject SD (wSD), absolute MDC, and relative MDC (MDC%) for each metric. MDC was calculated as 1.96×√2×wSD based on 3 repeated runs per time point. Results are presented as internal/external measurements. Lower MDC% indicates greater repeatability and sensitivity to detect true physiological change.

### Sensitivity of Optic Nerve Sheath Metrics to Postural Changes

Postural changes in 6 participants had a variable impact across metrics shown in Table 3. Internal sheath thickness was the most sensitive to posture, showing a significant +30.6/+31.5% increase from seated to raised and supine positions (ANOVA *P* = 0.01; seated vs raised *P* = 0.01), with 50% of participants showing a consistent upward trend. This was also observed for external thickness (+23.2%/+19.1%, *P* = 0.01). All other metrics changes were nonsignificant. Area and 3D diameter showed modest increases across positions, while 2D diameters changed minimally with posture. Uniformity decreased, and eccentricity varied. Supplementary video 5 (Supplemental Digital Content 5, http://links.lww.com/RLI/B78) illustrates observed changes in sheath thickness for a participant.

**TABLE 3:** Summary of Posture-dependent Changes for Internal and External Measurements With 6 Participants

Metric	Seated	Raised (Δ%)	Supine (Δ%)	*P* (ANOVA)	p_Seat↔Rais_	p_Rais↔Sup_
Internal metrics						
Diameter (2D)	5.26	5.37 (+2.1)	5.39 (+2.4)	0.85	—	—
Diameter (3D)	5.35	5.52 (+3.3)	5.62 (+5.1)	0.28	—	—
Area	22.26	23.68 (+6.4)	24.61 (+10.6)	0.26	—	—
Thickness	0.78	1.02 (+30.6)	1.03 (+31.5)	0.01	0.01	0.91
Uniformity	0.26	0.24 (–8.1)	0.23 (–12.1)	0.68	—	—
Eccentricity	0.57	0.59 (+3.8)	0.53 (–7.8)	0.61	—	—
External metrics						
Diameter (2D)	6.82	6.81 (–0.1)	6.80 (–0.3)	0.99	—	—
Diameter (3D)	6.57	6.76 (+3.0)	6.74 (+2.7)	0.48	—	—
Area	33.18	35.56 (+7.2)	35.30 (+6.4)	0.39	—	—
Thickness	1.32	1.62 (+23.2)	1.57 (+19.1)	0.01	0.01	0.61
Uniformity	0.43	0.36 (–16.0)	0.38 (–10.9)	0.32	—	—
Eccentricity	0.63	0.64 (+0.9)	0.57 (–9.8)	0.60	—	—

The table reports the mean values per posture, percent change from the seated baseline, and the results of statistical tests.

### Effects of 24-hour Normobaric Hypoxia

**FIGURE 6:**
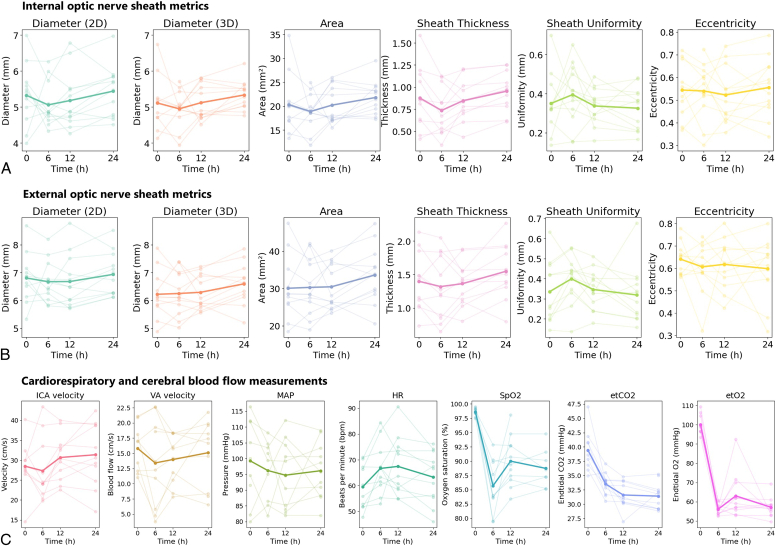
Each subplot shows individual participant trajectories (faint lines) and group-level means (bold lines) across timepoints (0, 6, 12, and 24 h) for 5 internal (A) and external (B) optic nerve sheath metrics. C, Shows the group-level means and individual plots of physiological parameters over time.

Figure 6 summarizes the optic nerve sheath metrics and physiological variables during 24-hour hypoxia exposure. At 24 hours, both internal and external measurements exhibited consistent increases from baseline, with the most prominent changes observed in area and sheath thickness. Internal area increased by 6.3% (*P* = 0.2) and internal sheath thickness by 8.2% (*P* = 0.04), while internal 2D diameter showed only a modest 1.8% rise (*P* = 0.9). External area increased by 11.2% (*P* = 0.3) and external sheath thickness by 10.4% (*P* = 0.2), while external 2D diameter increased by 1.8% (*P* = 0.9). The trend of the uniformity metric was opposite to that of diameter, with an initial increase followed by a decrease for both the internal and external measures. Eccentricity decreased over time for both internal (*P* = 0.7) and external (*P* = 0.8) measures. Mixed-effects models showed a significant effect of time for internal sheath thickness (*P* = 0.04), with post hoc comparisons indicating a significant increase between 6 hours and 24 hours. No other metrics showed statistically significant time effects. Across all models, MAP showed a consistent negative association with internal metrics, reaching significance for diameter (3D; *P* = 0.003), area (*P* = 0.003), and sheath thickness (*P* = 0.002). Sheath thickness was also associated with HR (*P* = 0.04, negative) and etCO_2_ (*P* = 0.03, positive). Correlation analyses between optic nerve sheath metrics and physiological parameters did not identify any statistically significant associations. However, the strongest positive trends were observed between internal area and ICA velocity (r = 0.9, *P* = 0.05) and internal sheath thickness and ICA velocity (r = 0.8, *P* = 0.07). External measurements showed similar trends (area: r = 0.7, *P* = 0.1; thickness: r = 0.7, *P* = 0.09). Notably, the spread between participants in 3D diameter at 6, 12, and 24 hours was smaller than that observed in 2D diameter.

## DISCUSSION

In this study, we introduced freehand 3D ultrasound as a practical bedside technique for imaging the optic nerve sheath. The major findings of this study were: (1) probe translation and rotation introduce substantial error in 2D ultrasound measurements of the optic nerve sheath, which can be mitigated using 3D processing techniques, (2) internal and external sheath thickness increased with changes in posture, consistent with responses reported using invasive methods,^21^ and (3) during 24 hours of hypoxia, sheath thickness initially decreased at 6 hours but subsequently increased in parallel with cerebral blood flow.

What factors contribute to inaccurate ONS quantification? While 2D ultrasound of the ONSD may be sensitive to markedly elevated ICP (>20 mm Hg),^22^ results across studies remain inconsistent. Experimental studies have been unable to identify subtle changes in ICP (12 to 20 mm Hg) by quantifying the ONSD, likely due to technical error. This moderate ICP range (12 to 20 mm Hg), however, is clinically important, as it typically warrants monitoring but not intervention. Our findings suggest that a substantial amount of variability is driven by probe misalignment rather than by the physiological stimulus. Despite all images meeting high-quality criteria,^10^ Figure 4 illustrates the measurement ambiguity introduced by the sheath’s noncircular geometry and the ambiguity in defining the optic disc landmark. Translations and pitch rotations in probe alignment relative to the optic nerve sheath caused diameter differences of up to 28%. From a practical perspective, this means that an individual with an ONSD of 4 mm could be quantified as anywhere from 2.8 to 4  mm, which, based on previous data highlighting a linear relationship between ICP and ONSD in pediatric neurocritical care patients,^23^ corresponds to an ICP prediction of 0 to ~25 mm Hg. While this example is extreme, it highlights why substantial variation exists in prior studies and why subtle changes in ICP have previously been difficult to quantify.

Three-dimensional freehand reconstructions across scan orientations yielded consistent morphology (Figs. 2–3 and supplementary video 4, Supplemental Digital Content 4, http://links.lww.com/RLI/B77). The eccentric shape of the optic nerve sheath corresponds well with the morphology observed in MRI data.^24^ The 3D diameter showed the lowest intraobserver and interobserver variability (<4%), comparable or superior to 2D diameter measurements. While sheath thickness and area were more variable (<14.4%), they proved more sensitive to physiological changes. Previously, more advanced morphologic^25,26^ and dynamical^27^ descriptors of the optic nerve sheath have been shown to improve sensitivity to changes in ICP. Freehand 3D ultrasound may be used in conjunction with these techniques and the quantification of sheath uniformity and eccentricity to further enhance the reliability and limits of detection to changes in ICP.

The optic nerve sheath is continuous with the brain’s subarachnoid space, along which elevated ICP is transmitted. It was hypothesized that ICP-related distension may not distribute evenly around the sheath’s circumference, predisposing to locoregional bulging or variability rather than a perfectly uniform expansion. Here, sheath uniformity captured a decreasing trend in angular variability and showed an inverse relationship to sheath thickness that warrants further investigation.

Is the optic nerve sheath sensitive to changes in posture in healthy individuals? Intracranial pressure is known to decrease in a mono-exponential decay during postural changes from supine to upright.^28,29^ During postural changes, internal and external sheath thickness increased by over 30% (*P* = 0.01) between the seated and head-raised (30 degrees) positions but did not change between the head-raised and supine positions. Typical postural changes alone can shift ICP by nearly an order of magnitude: in healthy adults, moving from supine (~10 to 12 mm Hg) to an upright seated or standing posture produces a drop of about 8 to 10 mm Hg, corresponding to a ~40% to 80% relative decrease depending on baseline.^21,30^ Notably, the observed changes in sheath thickness also align with previous CT-based reports of ~15% increases in sheath area when transitioning from upright to supine posture.^31^ The 2D Diameter showed minimal change. While we detected a clear change in sheath thickness between the upright and 30° head-up positions (corresponding to an expected ICP change ~5 mm Hg^28,29^), no metric changed between the 30 degrees head-up and supine positions, despite an expected ICP change of ~8 mm Hg.^28,29^ With only 5 minutes allowed for haemodynamic stabilization, the time frame may have been insufficient.

Is the optic nerve sheath sensitive to changes during 24 hours of exposure to hypoxia? Hypoxia induces vasodilation of cerebral arteries to maintain oxygen delivery. However, this occurs alongside ventilatory hypocapnia, which promotes cerebral vasoconstriction. The net effect on ICP depends on the balance between these opposing mechanisms. At moderate altitudes, ONSD may increase,^32,33^ though its relationship with acute mountain sickness remains uncertain.^34^ In the present study, both internal and external sheath thickness and area increased significantly after 24 hours of hypoxia, whereas 2D diameter did not change significantly over time. From a sensitivity standpoint, it is notable that sheath thickness and area decreased slightly at 6 hours and then rose progressively, paralleling changes in internal carotid artery velocity. Previous work has shown that sheath area correlates with ICP, especially when accounting for optic canal anatomy.^35,36^ Prior work also shows that ICP at rest may not increase under hypoxia on average, but susceptible individuals can experience marked elevations. MRI studies have demonstrated parenchymal swelling and venous compression after ~22 hours of hypoxia.^37^ Against this backdrop, the results suggest that the moderate hypoxia produced a moderate rise in ICP in the range of 12 to 20 mm Hg, and that sheath thickness was the only measure sensitive enough to capture this change. Because sheath thickness reflects both sheath distension and alterations in internal nerve size, it may provide a more comprehensive and anatomically grounded metric that is especially sensitive to ICP.

How confident can we be that the observed changes exceed measurement error? To address this, we calculated the MDC. In the posture experiment, the >30% increase in sheath thickness clearly exceeded MDC, providing strong evidence that postural change produces true morphometric adaptation that can be measured best with sheath thickness. Changes in area and 3D diameter were close to the MDC threshold, whereas 2D diameter did not approach it. The changes observed during hypoxia were more modest: increases in sheath thickness and area trended consistently upward but remained slightly below their respective MDC thresholds. Notably, inspection of individual time courses (Fig. 6) shows that 3D-derived metrics exhibit less within-subject fluctuation and more continuous trajectories. Taken together, these results suggest that the observed hypoxia-related changes in area and sheath thickness represent subtle but consistent physiological trends that, while modest in magnitude, are unlikely to reflect measurement error. This interpretation is reinforced by the high interobserver agreement demonstrated by ICC values, which supports the reliability of the measurements and strengthens confidence that the observed trends are physiologically meaningful.

What are the study limitations? The use of a commercial ultrasound system prevented access to raw radiofrequency data and synchronization of DICOM acquisition with the tracking device. Images were captured through an external card, which introduced bandwidth constraints and occasional loss of fidelity, including pixel dropout and poor delineation at the volume edges. Invasive ICP measurements were not performed; instead, we relied on posture, cardiorespiratory metrics, and hypoxia-related literature as physiological surrogates. Findings are based on a relatively small sample of 12 healthy volunteers. Probe movement and anatomic variability add uncertainty, as small shifts in nerve orientation can significantly affect imaging. The optic nerve sheath’s noncylindrical, asymmetric shape also complicates alignment to a true coronal plane, which may introduce geometric distortion.

What comes next? In future work, we will explore sensor-free 3D freehand scanning to further improve accessibility, matrix-array transducers for single-shot 3D imaging, and comparisons with invasive ICP to determine diagnostic sensitivity and specificity. Improving monitoring fidelity in the moderate ICP range (12 to 20 mm Hg), where management decisions are nuanced and reliable noninvasive trends are of high clinical value, should be a priority for future investigations. Using volumetric metrics, such as optic nerve sheath volume, rather than single-plane measurements from 2D or 3D assessments, should reduce alignment sensitivity; volume is less affected by local shape changes and enables measurements in patients who cannot cooperate. Freehand alignment variability may also contribute to plane measurement error, as the nerve is generally more in alignment with the image axis when 3D scanning is performed from the forehead to the chin compared with scans from the nose to the ear. Volumetric metrics are independent of the sweeping motion and therefore mitigate this problem.

In conclusion, our study demonstrates the feasibility of bedside optic nerve sheath assessment using freehand 3D ultrasound. Leveraging volumetric information, for example, optic nerve sheath thickness and area, provides a more robust and physiologically responsive evaluation of morphologic changes. Three-dimensional freehand ultrasound of the optic nerve sheath may provide greater sensitivity to subtle changes in ICP and offers advantages for noninvasive patient monitoring.

## Supplementary Material
